# A CT-Based Radiomic Signature Can Be Prognostic for 10-Months Overall Survival in Metastatic Tumors Treated with Nivolumab: An Exploratory Study

**DOI:** 10.3390/diagnostics11060979

**Published:** 2021-05-28

**Authors:** Valentina D. A. Corino, Marco Bologna, Giuseppina Calareso, Lisa Licitra, Mariagrazia Ghi, Gaetana Rinaldi, Francesco Caponigro, Franco Morelli, Mario Airoldi, Giacomo Allegrini, Alessandra Cassano, Daris Ferrari, Aurora Mirabile, Alicia Tosoni, Danilo Galizia, Marco Merlano, Andrea Sponghini, Gabriella Moretti, Luca Mainardi, Paolo Bossi

**Affiliations:** 1 Department of Electronics, Information and Bioengineering (DEIB), Politecnico di Milano, 20133 Milan, Italy; marco.bologna@polimi.it (M.B.); luca.mainardi@polimi.it (L.M.); 2 Radiology Department, Fondazione IRCCS Istituto Nazionale dei Tumori di Milano, 20133 Milan, Italy; giuseppina.calareso@istitutotumori.mi.it; 3 Head and Neck Medical Oncology Unit, Fondazione IRCCS Istituto Nazionale dei Tumori di Milano, University of Milan, 20133 Milan, Italy; lisa.licitra@istitutotumori.mi.it; 4 Department of Oncology and Hemato-Oncology, University of Milan, 20122 Milan, Italy; 5 Oncology 2 Unit, IRCCS Istituto Oncologico Veneto, 35128 Padua, Italy; mariagrazia.ghi@iov.veneto.it; 6 Medical Oncology Unit, Policlinico P. Giaccone University Hospital, 90127 Palermo, Italy; taniarinaldi02@gmail.com; 7 Head and Neck Medical and Experimental Oncology Unit, Istituto Nazionale Tumori, IRCCS Fondazione G. Pascale, 80131 Naples, Italy; f.caponigro@istitutotumori.na.it; 8 Department of Oncology, IRCCS Casa Sollievo della Sofferenza, 71013 San Giovanni Rotondo, Italy; franco.morelli@gemellimolise.it; 9 Medical Oncology 2 Unit, University Hospital “Città della Salute e della Scienza”, 10126 Turin, Italy; airoldim@yahoo.com; 10 Azienda USL Toscana Nord Ovest, 56121 Tuscany, Italy; giacomo.allegrini@uslnordovest.toscana.it; 11 Medical Oncology Unit, Policlinico Gemelli, 00168 Rome, Italy; alessandra.cassano@policlinicogemelli.it; 12 Medical Oncology Unit, San Paolo Hospital, 20142 Milan, Italy; daris.ferrari@asst-santipaolocarlo.it; 13 Medical Oncology Unit, San Raffaele Hospital, 20132 Segrate, Italy; mirabile.aurora@hsr.it; 14 Medical Oncology Department, Azienda USL/IRCCS Istituto delle Scienze Neurologiche di Bologna, 40139 Bologna, Italy; a.tosoni@ausl.bologna.it; 15 Multidisciplinary Outpatient Oncology Clinic, Candiolo Cancer Institute, FPO-IRCCS, 10060 Candiolo, Italy; danilo.galizia@ircc.it (D.G.); mcmerlano@gmail.com (M.M.); 16 Translational Research ARCO Foundation Cuneo, 12100 Cuneo, Italy; 17 “Maggiore della Carità” University Hospital, 28100 Novara, Italy; asponghini@libero.it; 18 GM Medical Oncology Unit, IRCCS Arcispedale S. Maria Nuova, 42123 Reggio Emilia, Italy; Gabriella.Moretti@ausl.re.it; 19 Medical Oncology, Department of Medical and Surgical Specialties, Radiological Sciences and Public Health University of Brescia, ASST-Spedali Civili, 25123 Brescia, Italy; paolo.bossi@unibs.it

**Keywords:** head and neck squamous cell carcinoma, radiomics, CT, overall survival

## Abstract

Baseline clinical prognostic factors for recurrent and/or metastatic (RM) head and neck squamous cell carcinoma (HNSCC) treated with immunotherapy are lacking. CT-based radiomics may provide additional prognostic information. A total of 85 patients with RM-HNSCC were enrolled for this study. For each tumor, radiomic features were extracted from the segmentation of the largest tumor mass. A pipeline including different feature selection steps was used to train a radiomic signature prognostic for 10-month overall survival (OS). Features were selected based on their stability to geometrical transformation of the segmentation (intraclass correlation coefficient, ICC > 0.75) and their predictive power (area under the curve, AUC > 0.7). The predictive model was developed using the least absolute shrinkage and selection operator (LASSO) in combination with the support vector machine. The model was developed based on the first 68 enrolled patients and tested on the last 17 patients. Classification performance of the radiomic risk was evaluated accuracy and the AUC. The same metrics were computed for some baseline predictors used in clinical practice (volume of largest lesion, total tumor volume, number of tumor lesions, number of affected organs, performance status). The AUC in the test set was 0.67, while accuracy was 0.82. The performance of the radiomic score was higher than the one obtainable with the clinical variables (largest lesion volume: accuracy 0.59, AUC = 0.55; number of tumoral lesions: accuracy 0.71, AUC 0.36; number of affected organs: accuracy 0.47; AUC 0.42; total tumor volume: accuracy 0.59, AUC 0.53; performance status: accuracy 0.41, AUC = 0.47). Radiomics may provide additional baseline prognostic value compared to the variables used in clinical practice.

## 1. Introduction

Recurrent and/or metastatic (RM) head and neck squamous cell carcinoma (HNSCC) have a dismal prognosis in cases when salvage surgery or reirradiation could not be offered. Traditionally, chemotherapy combination and targeted antiEGFR agent cetuximab have been the mainstay of treatment for RM HNSCC, with overall response rate at around 36% and median overall survival of 10.1 months [1]. With the advent of immunotherapy, the landscape of treatment opportunities has changed in HNSCC, with the approval by regulatory agencies of immune checkpoint inhibitors for RM disease, namely nivolumab and pembrolizumab. These agents have been shown to improve overall survival (OS) in comparison to standard treatment, both in relapsing disease resistant to cisplatin chemotherapy and in settings in which the disease is still benefitting from cisplatin, as therapeutic combination or alone [2,3]. This survival benefit has been obtained mainly with the contribution of the subgroup of patients experiencing long-term survival: in fact, the 1 and 2-year OS has almost doubled in respect to standard comparator treatments [2,3]. Therefore, the next logical step in clinical research is the discovery of factors defining which group of patients would benefit mostly from immune checkpoint inhibitors. This would allow, on the one hand, maximization of their use in RM HNSCC and, on the other, the study of new treatment opportunities for patients without any foreseen advantage from immunotherapy alone. As of today, only the PD-L1 combined positive score (CPS) has been identified as a tool to select patients who could benefit from the use of pembrolizumab, alone or with chemotherapy, in platinum-sensitive HNSCC patients. However, a positive CPS is present in about 85% of patients and its specificity is quite limited, so underlining the need for more research in this field. Radiomic biomarkers have been studied as a tool to predict the benefit of immune checkpoint inhibitors, mainly in non-small-cell lung cancer and melanoma [4]. In previous studies, radiomics has been used as a surrogate for either PD-L1 positivity [5,6] or RECIST response to immunotherapy [7,8,9,10,11,12], while only a few studies have used radiomics to develop prognostic models for survival [13].

In the current paper, we present the results of radiomic analysis on pre-treatment images of patients receiving nivolumab for RM HNSCC patients to predict 10-month survival.

## 2. Materials and Methods

### 2.1. Patient Population

We considered patients enrolled in the “Nivactor” clinical trial, a phase IIIb trial with nivolumab 240 mg, in subjects with RM HNSCC resistant to platinum-based chemotherapy. The primary objective of the trial was to assess the incidence of high-grade (grade 3 or higher), treatment-related, selected adverse events (AE); secondary objectives were to characterize the outcome of all AE, and to assess overall response rate, OS and Progression Free Survival (PFS). Main inclusion criteria of the trial were: histologically confirmed RM HNSCC (oral cavity, pharynx, larynx) not amenable to local therapy with curative intent; Eastern Cooperative Oncology Group (ECOG) performance status ≤2; tumor progression or recurrence within 6 months of last dose of platinum therapy in the adjuvant (i.e., with radiation after surgery), primary (i.e., with radiation or prior to it, or to surgery as induction chemotherapy) or RM setting; measurable disease by CT or MRI per RECIST 1.1 criteria [14].

Baseline clinical data were considered in the analysis (age, sex, performance status, type of recurrence, number of lesions and site of metastasis), as well as biological characteristics (HPV status, as identified by p16 immunochemistry expression, and PD-L1 expression, as assessed by IHC 22C3 pharmDx)

For the current radiomic analysis (an ad-hoc of the clinical trial, not part of the protocol), we selected patients enrolled in the “Nivactor” project who met the following inclusion criteria: (1) availability of baseline CT imaging; (2) follow-up of at least 10 months. Out of 127 patients enrolled in the trial, a subset of 85 patients was included in our study. The main clinical and follow-up data for the selected patients are displayed in Table 1. The 85 patients were divided into training and test sets based on the enrollment date, with the first 68 patients (80%) assigned to the training set and the remaining 17 (20%) assigned to the test set.

### 2.2. Image Acquisition

Contrast-enhanced CT images were acquired for each of the 85 patients. Images were acquired using 25 different scanners (see Supplementary Materials Table S1, for the full list) and different image acquisition parameters such as pixel spacing, slice thickness, tube voltage and tube current. Details of the CT image acquisition parameter are reported in Supplementary Materials Table S2.

### 2.3. Image Segmentation

Each tumor mass was segmented by an expert radiologist (G.C.) with more than 10 years of experience. An example of segmented images is displayed in Supplementary Materials Figure S1. In case of multiple tumor masses, only the largest was segmented.

### 2.4. Image Preprocessing

Image preprocessing was performed to reduce the imaging-related variability. First, a 3D Gaussian filter with a 3 × 3 × 3 voxel kernel and σ = 0.5 was used to denoise the images. Then, voxel size resampling to an isotropic resolution of 2 mm (as in [15]) was performed with B-spline interpolation.

### 2.5. Radiomic Features Extraction

The extraction of radiomic features was performed using Pyradiomics 3.0 [16]. A total of 536 features was extracted. The 536 features belonged to different categories: shape and size (14 features), first order statistics (18 features), textural (40 features), and wavelet (464 features). Textural features were computed using the grey level co-occurrence matrix (GLCM) and the grey level run length matrix (GLRLM). The full list of radiomic features is available in the Pyradiomics documentation [17]. A fixed-bin width histogram discretization (0.5 Hounsfield units per bin) was used prior to features extraction.

### 2.6. Clinical Endpoint

The clinical endpoint of interest for this exploratory ad-hoc radiomics study was overall survival (OS) at 10 months. OS was defined as the time (in days) between the beginning of treatment with nivolumab and the day of death or last contact. Moreover, we considered response to treatment according to RECIST 1.1 criteria [14].

### 2.7. Radiomic Signature Training Pipeline

**Figure 1:**
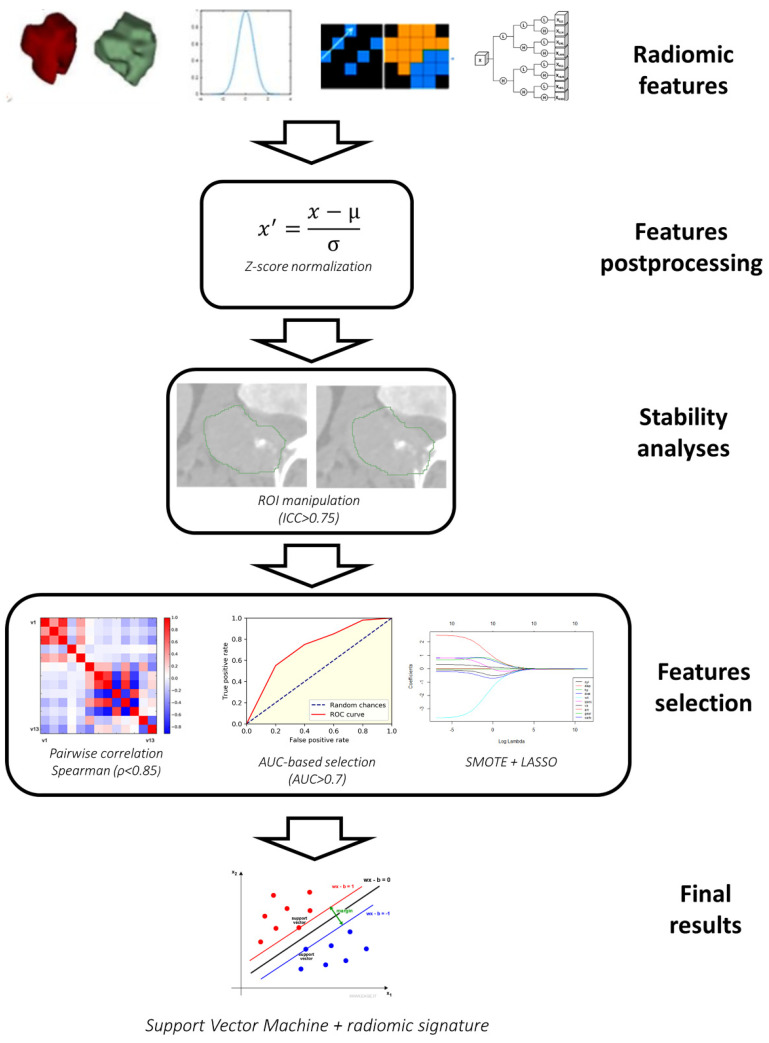
Workflow for the training of the radiomic signature.

Figure 1 shows the scheme, including all the steps for the training of the radiomic signature for survival classification.

The first postprocessing step was Z-score normalization, performed to ensure that the different features have similar ranges of values.

The normalized features underwent a series of feature selection steps that included the following: (1) selection of stable features; (2) selection of non-redundant features; (3) selection of features with high area under the curve (AUC) for the classification problem; (4) Least Absolute Shrinkage and Selection Operator (LASSO).

Only features that were stable to variations in the ROI were considered. Stability of features was assessed through small translations of the ROI (10% of the sizes of bounding box) as described in a previous study [18]. Stability was quantified using intra-class correlation coefficient (ICC) and features were considered stable if ICC was at least 0.75 [19].

Removal of redundant features was performed using pairwise correlation. When a pair of features had a Spearman correlation coefficient above 0.85 only one of the two was kept. In particular, only the one with lower mean correlation with all the other *n-2* features was selected.

Among the non-redundant features, only those with high classification performance were selected for the next stage. The classification performance of the features was quantified using the AUC, and only those with AUC > 0.7 were selected for the next step.

The final predictive model for 10-month survival prediction was developed using the least absolute shrinkage and selection operator (LASSO) in combination with support vector machine with cubic kernel [20]. LASSO is a type of model that depends on a tuning parameter λ, which controls the number of features used (the lower the λ, the higher the number of features). The optimal value for λ was selected through internal cross-validation of the training set. The features selected by LASSO were given as input to a support vector machine classifier, thus deriving the radiomic signature. Patients with signature higher than threshold were classified as high-risk (HR) patients, while the others were classified as low-risk (LR) patients. The optimal threshold was estimated using the Receiver Operating Characteristic (ROC) curve of the training set. In particular, the threshold corresponding to the point of the curve closer to the optimal point of the ROC curve (sensitivity 100%, false positive rate 0%) was selected. Prior to the training of the classifier, data balancing was performed using the Synthetic Minority Oversampling Technique (SMOTE) [21], a technique used to artificially oversample a class.

### 2.8. Radiomic Signature Validation

To validate the performance of the trained model, we used the 17 patients in the test set. The images of the test set underwent Z-score normalization (using the mean and standard deviation estimated in the training set) and the same features selected in the training set were isolated.

The SVM model was applied to obtain the radiomic signature for the patients in the test set, and a threshold was applied to obtain the predicted class. The numeric signatures and the predicted classes are used to compute the confusion matrix and the ROC curve, which are used to compute the AUC, and the accuracy, sensitivity and specificity of the signature.

### 2.9. Comparison of Radiomic Signature with Clinical Features

To provide a reference for the performance of the radiomic signature, the classification performance of baseline predictors used in the clinical practice (volume of largest lesion, total tumor volume, number of tumor lesions, number of affected organs, performance status, presence of non-metastatic tumor) was evaluated. As for radiomic signature, AUC, accuracy, sensitivity and specificity of the model were used as quality metrics.

### 2.10. Comparison with Volume

Since it is known that signatures obtained from radiomic features may present high correlation with simple geometrical features such as volumes, and therefore become useless, the Spearman’s correlation coefficient between ROI volume and the radiomic signature was computed.

## 3. Results

### 3.1. Radiomic Signature Training and Validation

The signature was composed of the following three features: original_shape_VoxelVolume, original-glrlm-RunLengthNonUniformity and waveletHLH-firstorder-Kurtosis.

**Figure 2:**
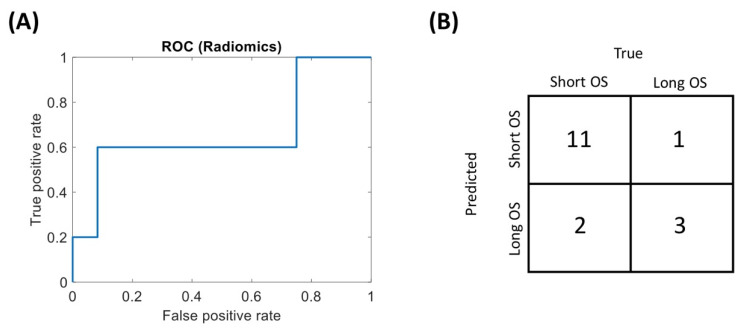
Performance of the radiomic signature on the test set. (**A**) Receiver operating characteristics (ROC) curve of the signature. (**B**) Confusion matrix.

Figure 2 shows the ROC curve and the confusion matrix for the radiomic-based classification, and the ROC curve of the radiomic signature in the test set. The binary radiomic classification reached an accuracy of 0.82 (0.59–0.94), a sensitivity of 0.60 (0.23–0.88) and a specificity of 0.92 (0.65–0.99) with an AUC of 0.67 (0.27–1) in the test set.

### 3.2. Comparison with Clinical Features

**Figure 3:**
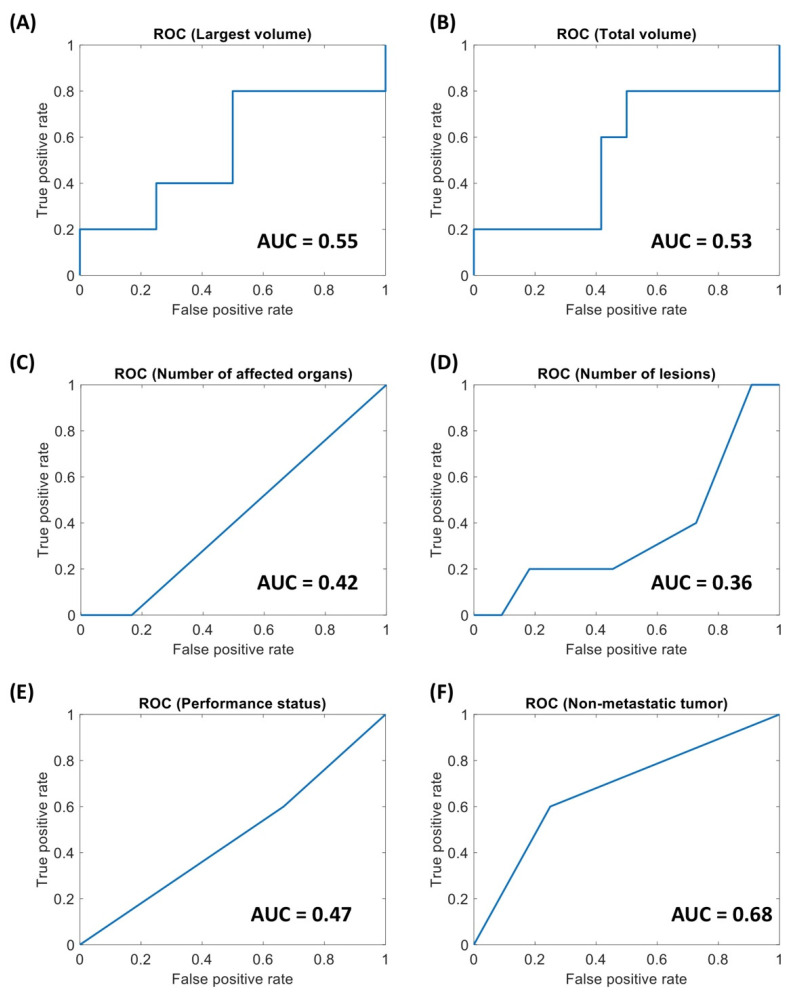
Receiver Operating Characteristics (ROC) curves for the clinical variables considered for the NIVACTOR project. (**A**) Volume of the largest Region Of Interest (ROI). (**B**) Total tumor volume. (**C**) Number of affected organs. (**D**) Number of lesions. (**E**) Performance status. (**F**) Non-metastatic status.

**Figure 4:**
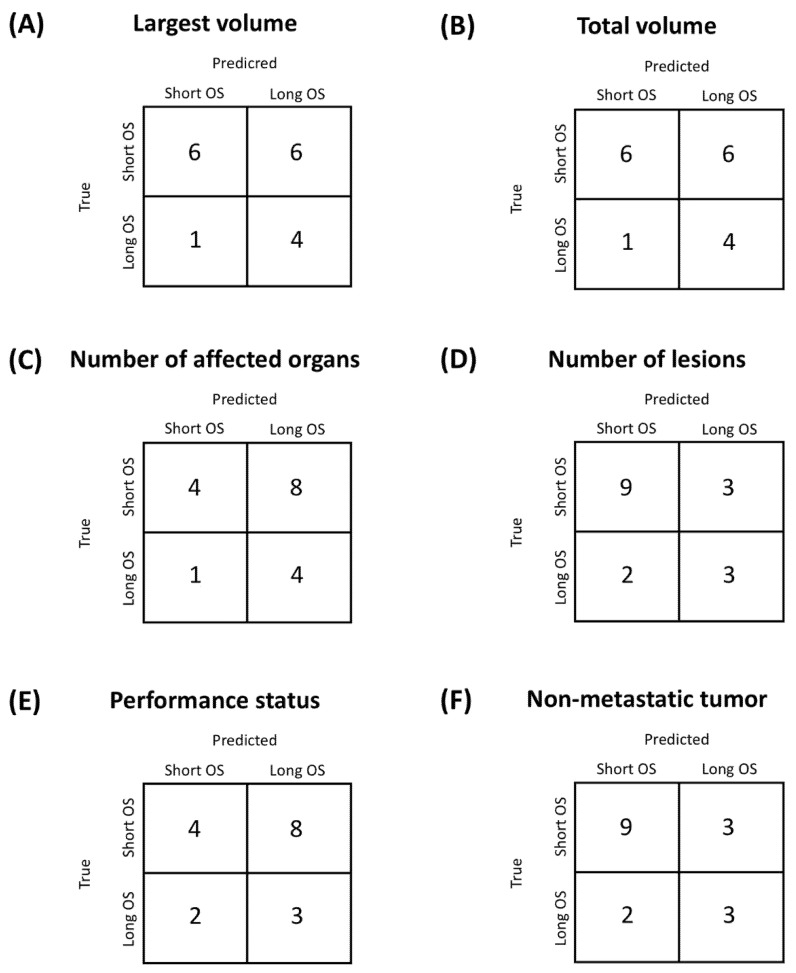
Confusion matrices for the clinical variables considered for the NIVACTOR project. (**A**) Volume of the largest Region Of Interest (ROI). (**B**) Total tumor volume. (**C**) Number of affected organs. (**D**) Number of lesions. (**E**) Performance status. (**F**) Non-metastatic status.

The ROC curves for the clinical variables, with the corresponding AUC, are displayed in Figure 3. Figure 4 displays the confusion matrices obtained using optimal threshold. Since for number of lesions and number of affected organs the ROC curves were strongly below 0.5, the direction of the thresholding was inverted (patients with less than two regions or affected organs are considered long survivors). Table 2 sums up the quality metrics obtainable from the confusion matrices in Figure 4. It can be observed that for all clinical variables the AUC is close to 0.5, i.e., random classification, highlighting that the clinical variables are not able to predict the prognosis of patients with HNSCC.

### 3.3. Comparison with Volume

**Figure 5:**
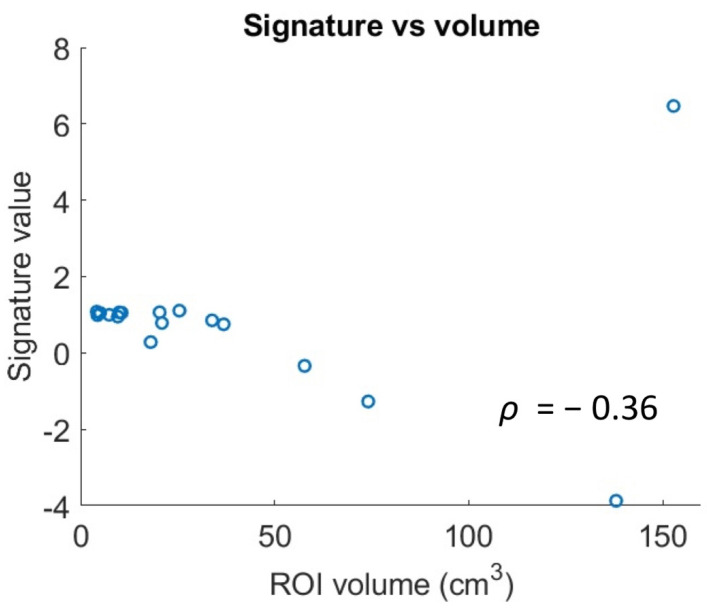
Scatterplot representing the correlation between the volume of the Region Of Interest (ROI) and the radiomic signature.

The scatterplot in Figure 5 displays the relationship between the volume of the ROI and the radiomic signature in the patients of the test set. The Spearman correction is weak and not significant (Spearman correlation coefficient ρ = −0.36, *p* = 0.15). Therefore, tumor volume only explains part of the variability of the radiomic signature.

## 4. Discussion

Statement of principal findings:

The main findings of this proof-of-concept study are: (i) radiomic features can predict long (>10 months) survival from baseline imaging in patients with recurrent and/or metastatic, platinum-resistant HNSCC treated with nivolumab; (ii) the predictive power of the radiomic signature is higher than that of any other clinical variables acquired before immunotherapy treatment.

Meaning of the study: possible mechanisms and implications for clinicians:

The radiomic model was obtained after proper feature selection by using a support vector machine with a cubic kernel. The three features that were used to train the model were the following: original-shape-VoxelVolume, original-glrlm-RunLengthNonUniformity and waveletHLH-firstorder-Kurtosis.

A low value of original-glrlm-RunLengthNonUniformity is associated with runs that are equally distributed along run lengths [22], so a higher value of the feature results in runs of a particular length that are more frequent than others. To get a better interpretation of the features it may be considered that, in this dataset, original-glrlm-RunLengthNonUniformity is positively correlated with original-glrlm-GreyLevelNonUniformity (Spearman’s correlation coefficient 0.91, *p* = 1.13 × 10^−27^), which has been used in the past to describe intra-tumoral heterogeneity [23]. The waveletHLH transform highlights the high frequencies (high pass filter) in the x and z direction and the low frequencies (low pass filter) in the y direction and kurtosis measures the peakedness of the intensity distribution [22]; therefore, a higher waveletHLH-firstorder-Kurtosis indicates a higher peakedness of the grey values in the waveletHLH of the original image. Such a feature is also positively correlated with original-glrlm-GreyLevelNonUniformity (Spearman’s correlation coefficient 0.57, *p* = 1.06 × 10^−8^), so the higher the feature, the higher the intra-tumor heterogeneity. Moreover, kurtosis has been previously used in in prognostic models for OS [13,24]. Finally, tumor volume has been shown to be a prognostic factor for OS [25] and so it is reasonable that tumor volume is an important feature in determining survival class (long/short). However, the results show that the volume of the largest ROI alone is not enough to obtain good results on the test set. The radiomic score contains an added value compared to volume alone, which is evident from the better classification results and by the low correlation of tumor volume and the radiomic score.

It is possible to see that the radiomic score on which the classification is based is the feature with the best predictive performance among those obtainable at baseline (AUC = 0.67, accuracy 0.82). Of the clinical features, the number of lesions has an AUC much lower than 0.5 (AUC = 0.36), and therefore using an inverted threshold (in this case *n* ≤ 2) also provides good prognostic accuracy, with the same sensitivity but lower specificity compared to the radiomic features. The number of affected organs has an AUC = 0.58, but the selected threshold leads to a low accuracy (0.41). The other three clinical variables had low AUC and accuracy.

Strengths and weaknesses of the study:

The preliminary results seem to suggest that radiomics may provide a stronger tool for prediction of long-term survival of patients treated with nivolumab using only information available at baseline (i.e., medical imaging). The prediction of the benefit of immune checkpoint inhibitors in HNSCC appears to be challenging, as clinical features seem not to be predictive. Therefore, this study, even if only preliminary, may be a first step in better understanding response to immunotherapy and thus prognosis of patients with HNSCC.

A limitation of the study is the limited size of the population, which makes the study only a proof-of-concept, requiring further validation sets.

Strengths and weaknesses in relation to other studies, discussing particularly any differences in results:

This was not the first study in which radiomics has been applied to define the prognosis of patients treated with immunotherapy [5,7,8,9,12,13,14,15,16,17,18,19,20,21,22,23,24,25,26], even though most of the studies refer to lung cancers [5,7,8,12]. In most of these studies, the developed models were referred to RECIST response, but in [12] the predictive power for long term OS (15 months) was investigated starting from PET features. Our study investigates the performance of CT imaging, which is more frequently used and could provide additional information.

In this regard, it should be underlined that immunotherapy in cancer patients obtains its advantages mostly by increasing long-term survival; this is particularly evident in HNSCC, where the use of immune checkpoint inhibitors formed a plateau in the survival curves, which has not been previously reached with chemotherapy and targeted agents. Moreover, immunotherapy could prime the tumoral microenvironment for response to further chemotherapy lines, and consequently obtain increased survival [27].

Unanswered questions and future research:

So far, we lack clinical and biological factors able to predict the benefit of immune checkpoint inhibitors in HNSCC, except for performance status and PD-L1 status, even if the role of the latter is confirmed only in patients treated with pembrolizumab, with or without chemotherapy, in platinum-sensitive diseases. Therefore, when validated in larger series, CT-radiomics can provide an additional source of information that may help the prognostic performance for OS. Moreover, possible future developments of radiomics could include the development of a non-invasive characterization of tumor microenvironment [28] to provide better tumor characterization and exploit the information in predictive and prognostic models.

## 5. Conclusions

In this proof-of-concept study radiomic features were able to predict long/short survival (>10 months) at baseline. The predictive power of the radiomic features was found to be higher than that of any other variables acquired before immunotherapy treatment. Although preliminary, the results of this study may be a first step in a path that leads to the use of CT-radiomics in clinical practice.

## Figures and Tables

**Table 1:** Clinical data of patients involved in the study and best response to treatment. Numerical variables are expressed as median and inter-quartile range.

Patients Clinical Data (N = 85)	
Age at diagnosis (years)	63 (57–70)
Sex	Female: 17 (20%)
	Male: 68 (80%)
Performance status	Status 0: 27 (32%)
	Status 1: 55 (65%)
	Status 2: 3 (3%)
PD-L1 expression	Positive: 29 (34%)
	Negative: 33 (39%)
	Unknown: 23 (27%)
HPV status	Positive: 12 (14%)
	Negative: 12 (14%)
	Unknown: 61 (72%)
Type of recurrence	Local: 9 (11%)
	Regional: 6 (7%)
	Loco-regional: 22 (26%)
	Distant alone: 19 (22%)
	Distant + other: 29 (34%)
Number of lesions	3 (2–4)
Number of affected organs	2 (1–3)
RECIST response	Progressive disease: 55 (65%)
	Stable disease: 17 (20%)
	Partial response: 11 (13%)
	Complete response: 1 (1%)
	Unknown: 1 (1%)

**Table 2:** Performance of the different variables in the prediction of long term survival (OS > 10 months).

Quality Metrics for Classification			
Variable	Accuracy	Sensitivity	Specificity
Radiomics	0.82 (0.59–0.94)	0.60 (0.23–0.88)	0.92 (0.65–0.99)
Largest volume	0.59 (0.36–0.78)	0.80 (0.38–0.96)	0.50 (0.25–0.75)
Total volume	0.59 (0.36–0.78)	0.80 (0.38–0.96)	0.50 (0.25–0.75)
Number of lesions	0.71 (0.47–0.87)	0.60 (0.23–0.88)	0.75 (0.47–0.91)
Number of affected organs	0.47 (0.26–0.69)	0.80 (0.38–0.96)	0.33 (0.14–0.61)
Performance status	0.41 (0.22–0.64)	0.60 (0.23–0.88)	0.33 (0.14–0.61)
Non-metastatic tumor	0.71 (0.47–0.87)	0.60 (0.23–0.88)	0.75 (0.47–0.91)

## Data Availability

The data presented in this study are available on request from the corresponding author. The data are not publicly available due to restrictions due to privacy issues.

## Supplementary Materials

The following are available online at https://www.mdpi.com/article/10.3390/diagnostics11060979/s1. Table S1, Distribution of the different scanners used in the study. Table S2, Image acquisition parameters for the computed tomography (CT) images used in the studies. Numeric values are expressed as median and inter-quartile ranges. Figure S1, Example of computed tomography image with the segmented tumor.
