# Supplementary material for: A CT-Based Radiomic Signature Can Be Prognostic for 10-Months Overall Survival in Metastatic Tumors Treated with Nivolumab: An Exploratory Study
Source: Diagnostics (Basel). 2021 May 28;11(6):979. doi: 10.3390/diagnostics11060979 (PMC8229740; doi:10.3390/diagnostics11060979)
Supplement: Supplementary file 1 [file diagnostics-11-00979-s001.zip › diagnostics-1235553-supplementary.pdf]

# Supplementary material: A CT-based radiomic signature can be prognostic for 10-months overall survival in metastatic tumors treated with nivolumab

**Valentina D.A. Corino [1,*], Marco Bologna [1], Giuseppina Calareso [2], Lisa Licitra [3,4], Mariagrazia Ghi [5], Gaetana Rinaldi [6], Francesco Caponigro [7], Franco Morelli [8], Mario Airoldi [9], Giacomo Allegrini [10], Alessandra Cassano [11], Daris Ferrari [12], Aurora Mirabile [13], Alice Tosoni [14], Danilo Galizia [15], Marco Merlano [15,16], Andrea Sponghini [17], Gabriella Moretti [18], Luca Mainardi [1] and Paolo Bossi [19]**

**Table S1.** Distribution of the different scanners used in the study.

| Scanner_Model | Scanner_Vend | Acquired_Patients_Numb |
|---|---|---|
| BrightSpeed | GE | 2 |
| Discovery CT 750 HD | GE | 1 |
| LightSpeed Pro 32 | GE | 1 |
| LightSpeed RT 16 | GE | 1 |
| LightSpeed VCT | GE | 6 |
| Optima CT 540 | GE | 3 |
| Optima CT 660 | GE | 7 |
| Revolution Evo | GE | 4 |
| Eclos | Hitachi | 3 |
| Scenaria | Hitachi | 1 |
| Brilliance 16 | Philips | 8 |
| Brilliance 40 | Philips | 1 |
| Brilliance 64 | Philips | 7 |
| iCT 128 | Philips | 3 |
| iCT 256 | Philips | 3 |
| Ingenuity | Philips | 2 |
| Biograph 16 | Siemens | 1 |
| Biograph 6 | Siemens | 1 |
| Sensation 64 | Siemens | 6 |
| Somatom Definition AS | Siemens | 2 |
| Somatom Definition AS+ | Siemens | 2 |
| Somatom Definition Flash | Siemens | 8 |
| Somatom Force | Siemens | 1 |
| Somatom Perspective | Siemens | 3 |
| Aquilion | Toshiba | 8 |

**Table S2.** Image acquisition parameters for the computed tomography (CT) images used in the studies. Numeric values are expressed as median and inter-quartile ranges.

| CT IMAGE ACQUISITION PARAMETERS | |
|---|---|
| **Tube Voltage (kVP)** | 120 [120-120] |
| **Tube current (mA)** | 197 [121-297] |
| **Slice thickness (mm)** | 2.5 [1.5-3] |
| **Pixel spacing (mm)** | 0.51 [0.50-0.82] |

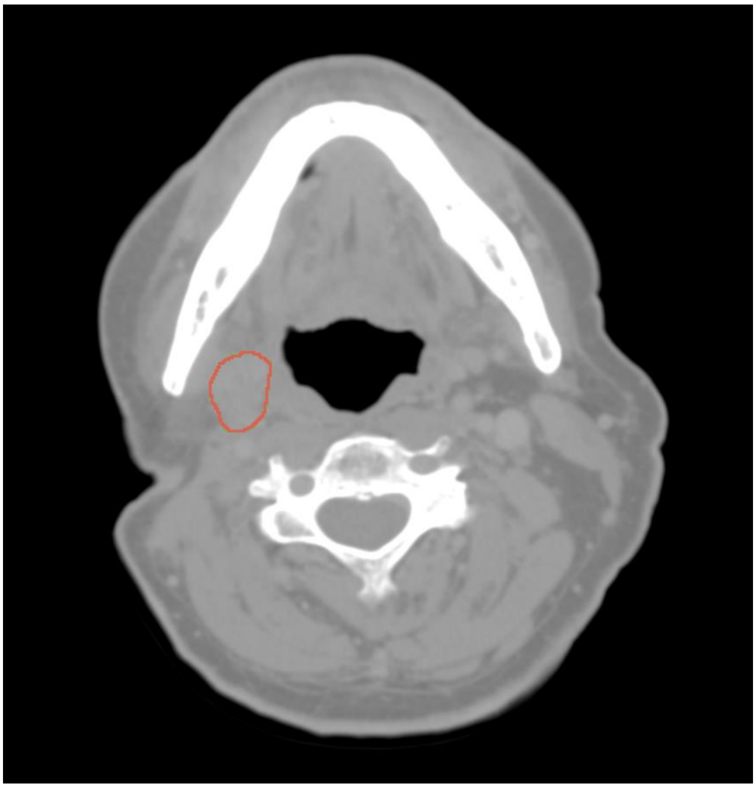

**Figure S1.** Example of computed tomography image with the segmented tumor.
